# Plasma Ammonia Levels Over the Course of a Hospitalisation for Overt Hepatic Encephalopathy

**DOI:** 10.1111/liv.70365

**Published:** 2025-10-03

**Authors:** Davide Erminelli, Chiara Mangini, Dario Gapeni, Lisa Zarantonello, Sara Montagnese

**Affiliations:** ^1^ Department of Medicine University of Padova Padova Italy; ^2^ Chronobiology Section, Faculty of Health and Medical Sciences University of Surrey Guildford UK

**Keywords:** cirrhosis, cognitive decline, hepatic coma, hospital stay, hyperammonemia, psychoactive drugs

## Abstract

**Background & Aims:**

The role of ammonia in the assessment of Overt Hepatic Encephalopathy (OHE) remains debated. The aim of the present study was to assess the time course of ammonia levels in relation to the severity and duration of an episode of OHE requiring hospitalisation.

**Methods:**

104 patients discharged between January 2021 and July 2024 with a diagnosis of OHE were included [80 males, 72 ± 11 years]. Clinical and laboratory indices, including ammonia levels prior to/during/after the hospitalisation, were recorded, along with the duration of the hospitalisation, precipitants, ammonia‐lowering and psychoactive treatment, where applicable.

**Results:**

Over the studied time frame, 58 (56%) patients had single OHE hospitalisations, while 20 (19%) had 2–7 OHE hospitalisations. Of the 104 hospitalisations, 30/63/7% were for OHE grades II/III/IV, respectively. Of the 58 single hospitalisations, 40/57/3% were for OHE grades II/III/IV, respectively. Ammonia levels increased significantly with increasing OHE grade; those prior to admission/after discharge were significantly lower than those on admission. Hospitalisations for grade IV OHE were longer. Ammonia levels on admission were lower in patients who were not fully recovered from OHE on discharge and those on chronic treatment with psychoactive drugs. All the above held true when analyses were restricted to the 58 single hospitalisations.

**Conclusions:**

Ammonia levels correlated with OHE presence and severity, and with the duration of hospitalization. Incomplete recovery and chronic treatment with psychoactive drugs were associated with lower ammonia levels.

**Summary**

- Hepatic encephalopathy (HE) is a neurological complication of advanced liver disease which heavily affects the quality of life of patients and their caregivers.
- The usefulness of measuring blood ammonia to support a diagnosis of HE is debated.
- Here we show how ammonia increases during an episode of HE requiring hospitalisation, and how its levels correlate with both the severity of the HE episode and the length of hospitalisation.
- Our data support the use of blood ammonia measurement to help HE diagnosis and management.

## 1 Introduction

The current definition of HE (i.e., a form of brain dysfunction caused by liver insufficiency and/or portal systemic shunt) [1] refers to pathophysiology, as in order to be qualified as hepatic, an acute encephalopathy in a patient with liver disease requires ammonia elevation [1]. Based on the above definition, ammonia is utilised for its negative predictive value. In addition, ammonia has also been shown to correlate with the severity of HE [2, 3], to predict liver‐related hospitalisations [4] and mortality [5]. Normal ammonia values suggest that hepatic failure/shunts are not severe enough to determine an increase in ammonia to a pathological range. Consequently, additional or other causes of altered mental status should be sought for [6].

However, the relationship between ammonia levels and mental status at any given time is affected by several confounders and by the temporal mismatch between plasma ammonia levels and both cerebral ammonia levels and the duration of their effect, on which limited information is available in humans. In further detail, based on the pathophysiology of the cerebral effects of hyperammonaemia (i.e., increased glutamine production in astrocytes, resulting in astrocyte swelling and alterations in neurotransmission [7]) may lag after plasma ammonia levels have lowered or normalised. In addition, the presence of hyperammonaemia does not implicate the expression of the HE phenotype, suggesting considerable inter‐individual sensitivity to ammonia, for example in relation to age [8], additional risk factors for cognitive impairment [9] and drugs [10].

In a recent study by Desplats et al. [10], four different cohorts of cirrhotic patients were split by the presence/absence of both acute encephalopathy and hyperammonaemia; HE was defined by the presence of both alterations. The study showed how approximately a third of patients with cirrhosis and encephalopathy were not hyperammonaemic, and they were exposed to significantly more medications compared to OHE patients. Hyperammonaemia was common also in patients with no encephalopathy, confirming it does not necessarily result in the OHE phenotype [11].

By contrast, in a 2019 retrospective study, Haj and Rockey [12] evaluated the relationship between ammonia levels and oral lactulose dosage in patients with cirrhosis and HE. They observed that total lactulose dose did not differ in relation to ammonia levels, which could be easily explained by the fact that ammonia levels had not been utilised to direct clinical decisions. In addition, the background hypothesis that lactulose dose should be titrated based on ammonia levels is questionable, as response to lactulose is also extremely variable, and it is therefore recommended that it is titrated based on obtained bowel movements. Moreover, in a recent study, the usefulness of ammonia assessment was questioned based on the lack of correlation between ammonia levels and both the severity and the time to resolution of an OHE episode in a group of 44 inpatients [13]. However, this study was based on a solely phenotypical definition of OHE and was also confounded by described ammonia measurement issues such as variability in sample handling and processing, which are less common in Europe.

The aim of the present study was to assess the time course of ammonia levels during an OHE‐related hospitalisation in relation to OHE duration/severity and the length of hospitalisation.

## 2 Patients and Methods

Retrospective Ethics Committee permission was obtained to review Diagnosis Related Group (DRG) discharge codes of clinical records of the Liver Unit, Internal Medicine Ward 5, Padova University Hospital (Italy) between January 2021 and July 2024. Patients with Hepatic Encephalopathy/Coma (DRG 572.2) as their first or second discharge code diagnosis were selected (regardless of ammonia levels on admission) and pertinent electronic hospitalisation records accessed to retrieve the following information: demographics, cirrhosis aetiology, presence of ascites, spontaneous/surgical portal‐systemic shunts, oesophageal varices, hepatocellular carcinoma, risk factors/any degree of neuropsychiatric impairment (e.g., from cerebrovascular disease or other neurological disorders). The first inpatient laboratory indices, either from bloods taken in the Accident and Emergency department or in the ward were recorded, including: full blood count, renal function, sodium, potassium, transaminases, gamma‐glutamine transferase, alkaline fosfatase, bilirubin, international normalised ratio (INR), albumin, glucose, C reactive protein, procalcitonin, thyroid stimulating hormone (TSH) and urine analysis. The Child‐Pugh score, Model for End‐stage Liver Disease (MELD) and Model for End‐stage Liver Disease‐Sodium (MELD‐Na) were also calculated and length of hospitalisation recorded.

During the hospitalisations, patients were looked after by one of 15 consultants (together with a number of varying level specialist trainees) amongst whom 12 are also hepatologists, and one (author SM) has a research interest in HE.

### 2.1 Characterisation of the OHE Episode

The following information was obtained: OHE grade on admission (according to Italian guidelines [14] and as fully detailed in [15]) and on any subsequent evaluation, precipitant (where identified and amongst infection, gastrointestinal bleeding, dehydration/diuretic overdose, electrolyte disorder and constipation [1]), duration of the HE episode (please see below for the definition of “resolution”), previous OHE history. A detailed record of treatment prior to, during hospitalisation and on discharge was obtained, with a particular focus on management of the precipitant, ammonia‐lowering/anti‐HE treatment (i.e., lactulose/lactitol, other laxatives, oenemas, rifaximin, branched chain amino acids, probiotics) and psychoactive drugs (i.e., benzodiazepines, opioids or their derivatives, antidepressants, antipsychotics). Based on information retrieved from the hospital notes and in line with Italian guidelines [14], the OHE episode was qualified as resolved when the patient was oriented to time and space, presented normal mental status (i.e., not described as confused, agitated or somnolent) and had no flapping tremor.

### 2.2 Time‐Course of Plasma Ammonia Levels

In Padova University hospital, venous ammonia is measured in the emergency lab, with a turn‐around of 60–90 min; it is required that the EDTA tube reaches the laboratory in ice and plasma is separated immediately after arrival; the threshold for abnormality is 72 μmol/L.

The following plasma ammonia values were sought for:

- baseline, within the 6 months prior to the admission, where available from outpatient evaluations (i.e., not on the occasion of other admissions for OHE or for other reasons);
- T0, i.e., on admission, the first sample within the OHE hospitalisation, either from the Accident and Emergency department or from the ward;
- T1 to Tn, i.e., any subsequent measurement taken in the ward during the hospitalisation. As these were “unplanned” and dictated by clinical needs, they were analysed both in order and also taking into account of their time from T0 above, expressed in hours.
- post‐discharge, within the 6 months after discharge, where available from outpatient evaluations (i.e., not on the occasion of other admissions for OHE or other reasons for measurement).

### 2.3 Statistical Analyses

Results are expressed as mean ± SD or as count/percentage, as appropriate. Normality was tested for by the Shapiro–Wilk's test. Patient groups were compared by the Student t/Mann–Whitney *U* test or ANOVA/Kruskal‐Wallis ANOVA (post hoc Tukey test and Median test, respectively), as appropriate. Measures over time were compared by repeated measures ANOVA (post hoc Tukey test) or Friedman ANOVA, as appropriate. Differences between proportions were tested for by Pearson's chi‐square or Spearman ranks test. Analyses were carried out by the package Statistica, version 13.1 (Dell, Round Rock, TX).

## 3 Results

Over the convenience period January 2021 and July 2024, 5432 discharge records were produced. Of these, 107 (2%) had Hepatic Encephalopathy/Coma (DRG 572.2) as first (*n* = 57) or second (*n* = 50, with the first code being cirrhosis with or without alcohol misuse) discharge code and were therefore analysed in detail. After accurate revision, a further 3 were excluded as the DRG code had obviously been assigned incorrectly. Of the final 104 records, 38 (36.5%) belonged to patients admitted for OHE once, while the remaining 66 (63.5%) belonged to 20 patients (“repeats”) admitted for OHE for up to 7 times over the period of observation; thus, of the 104 records, 58 (56%) were single OHE records.

Demographic, clinical and laboratory indices are presented, by record and patient group, in Table 1. Procalcitonin, TSH and urine analyses were available in a relatively small proportion and were not analysed further.

**TABLE 1:** Demographic, clinical and laboratory indices, by record and by patient group.

	All records (*n* = 104)	First OHE records (*n* = 58)	Single OHE patients (*n* = 38)	Multiple OHE patients (*n* = 20)
Sex [males, *n* (%)]	80 (77)	48 (83)	33 (87)	15 (75)
Age (mean ± SD)	72 ± 11	70 ± 10	69 ± 9	72 ± 12
Aetiology [viral, alcohol, metabolic, other (%)]	15/31/14/40	14/36/9/41	13/42/5/40	15/25/15/45
Pugh (mean ± SD)	9.6 ± 2.0	9.6 ± 1.9	10.0 ± 1.7	8.9 ± 2.2
Child [A, B, C (%)]	3/56/41	4/55/41	3/50/47	5/65/30
MELD (mean ± SD)	16.8 ± 5.2	16.7 ± 5.3	17.5 ± 4.9	15.2 ± 6.0
MELD‐Na (mean ± SD)	18.9 ± 5.5	19.1 ± 5.8	20.2 ± 5.05	17.2 ± 6.7
TIPS [*n* (%)]	7 (7)	5 (9)	4 (11)	1 (5)
Spontaneous portal‐systemic shunt [*n* (%)]	42 (40)^a^	22 (38)	14 (37)	8 (40)
Previous OHE episodes [*n* (%)]	84 (80)^b^	38 (66)	25 (66)	13 (65)
OHE grade II/III/IV [*n* (%)] on admission	31/66/7 (30/63/7)	23/33/2 (40/57/3)	15/22/1 (39/58/3)	8/11/1 (40/55/5)
Precipitant [not identified/dehydration/constipation/infection/GI bleeding/mixed (%)]	23/17/12/10/1/37	29/17/14/9/2/29	34/16/13/11/0/26	20/20/15/5/5/35

^a^ Information available in 60 records.

^b^ Information available in 100 records.

No significant differences were observed between patients with one or more OHE episodes over the observation period. Overall, 31 hospitalisations (30%) were for grade II, 66 (63%) for grade III and 7 (7%) for grade IV OHE. The same proportions were reflected in the single OHE record subset (*n* = 58), with 23 hospitalisations (40%) for grade II, 33 (57%) for grade III and 2 (3%) for grade IV. In 24 (23%) hospitalisations the precipitant factor was not identified, while the most commonly identified precipitants were mixed, dehydration and constipation. Again, proportions were similar when analyses were restricted to the single OHE record subgroup.

The length of hospitalisation tended to be longer in patients with grade IV OHE, and the difference was significant in the comparison with patients with grade III (grade II 6.7 ± 5.1 days, grade III 5.8 ± 4.8 days, grade IV 12.3 ± 6.5 days; post hoc comparison grade III vs. grade IV: *p* < 0.01); the main effect was not confirmed when the analysis was confined to the single OHE record subset, which, however, only encompassed two patients with grade IV OHE. Patients were on a higher number of ammonia‐lowering drugs during hospitalisation (3.0 ± 0.9) and on discharge (2.6 ± 1.1) compared to on admission (2.1 ± 1.3), which was also confirmed in the single OHE record subgroup (on admission: 1.5 ± 1.1, during hospitalisation 2.9 ± 1.0, on discharge 2.3 ± 1.1).

**FIGURE 1:**
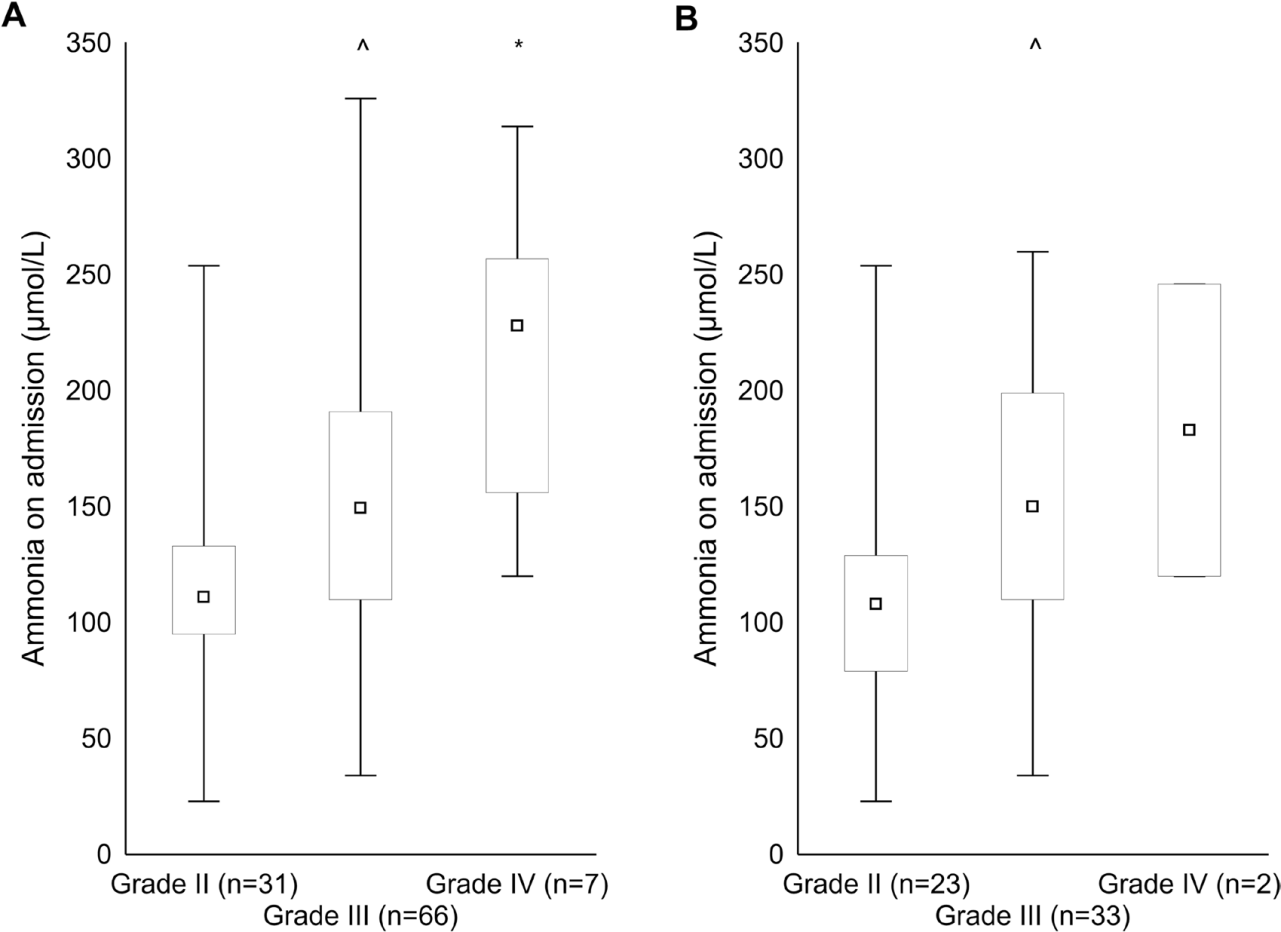
Ammonia levels [median, upper and lower quartile (box), range (whiskers)] on admission in individuals with OHE of varying severity in full record set (*n* = 104, panel A) and in the single OHE record set (*n* = 58, panel B). *^p* < 0.05 grade II vs grade III; *p < 0.01 grade II vs grade IV.

**FIGURE 2:**
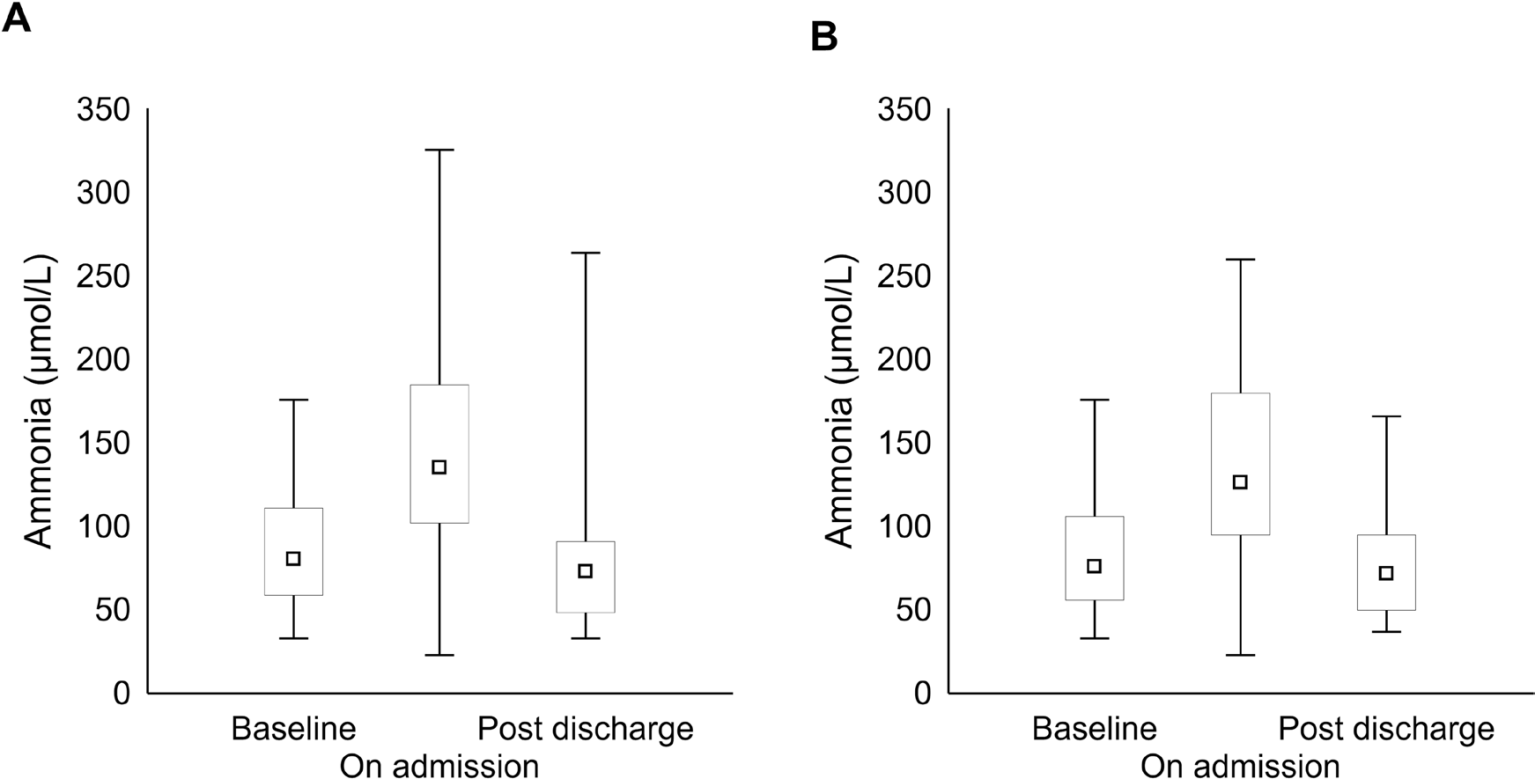
Ammonia levels [median, upper and lower quartile (box), range (whiskers)] prior to, on admission, and after hospitalisation in available patients from the whole record set (*n* = 27, panel A) and from the single OHE record subset (*n* = 18, panel B). Main effect significant with *p* < 0.0001 for panel A and *p* < 0.01 for panel B.

**FIGURE 3:**
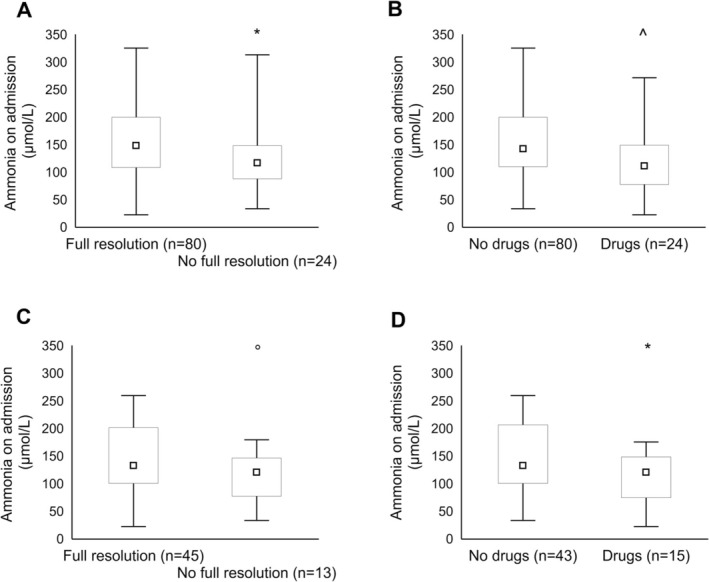
Ammonia levels [median, upper and lower quartile (box), range (whiskers)] in patients who had/had not fully recovered from the OHE episode on discharge and patients who were/were not on chronic psychoactive medication in the whole record set (panels A and B) and in the single OHE record subset (panels C and D). *p < 0.05, ^*p* < 0.01, 0.05 < °*p* < 0.1.

Ammonia levels increased progressively and significantly with increasing OHE grade both in all records (grade II: 118 ± 47 umol/L vs. grade III: 155 ± 62 umol/L vs. grade IV: 219 ± 65 umol/L; post hoc comparisons significant for grade II vs. grade III and IV; Figure 1A) and in the single OHE subgroup (grade II: 115 ± 52 umol/L vs. grade III: 153 ± 63 umol/L vs. grade IV: 183 ± 89 umol/L; post hoc significant for grade II vs. grade III; Figure 1B). Ammonia was measured at least once after admission in the majority of patients [92%, most commonly within the first 36 h of hospitalisation (Table 2)] and decreased significantly compared to ammonia on admission (*p* < 0.0001); subsequent measurements over the hospitalisation were obtained in varying proportions of patients and appeared to remain substantially stable (Table 2). Ammonia levels on admission were significantly higher than ammonia levels over the previous and the subsequent 6 months (Figure 2A); this was confirmed when the analysis was confined to the single OHE record subgroup (Figure 2B). Ammonia levels on admission were significantly lower in patients who were subsequently discharged not having fully recovered from the OHE episode (Figure 3A and Figure S1A); these patients were older (81 ± 9 vs. 70 ± 10 years, *p* < 0.0001) and more commonly qualified as having risk factors/some degree of neuropsychiatric impairment (59 vs. 17%, χ^2^ = 5, *p* < 0.05). Similarly, ammonia levels on admission were significantly lower in patients who were on chronic psychoactive medication (Figure 3B and Figure S1B). This held true when analyses were adjusted for the presence of infection and liver function (MELD); MELD, but not infections, was also significant on multivariable analyses. The relationship between ammonaemia and both incomplete OHE resolution and chronic psychoactive medication was confirmed when the analysis was confined to the single OHE record subgroup (Figure 3C,D).

**TABLE 2:** Ammonia levels and time of measurement, in hours from first measurement (i.e., T0, on admission).

	*n*	Ammonia (umol/L; mean ± SD)	Time from T0 (hours; mean ± SD)
T0 (on admission)	104	148 ± 63	0
T1	96	80 ± 30	27 ± 26
T2	65	80 ± 39	63 ± 33
T3	37	79 ± 42	120 ± 60
T4	22	86 ± 44	149 ± 55
T5	17	77 ± 45	196 ± 65
T6	8	71 ± 50	223 ± 71
T7	4	62 ± 36	256 ± 86
T8	4	62 ± 25	310 ± 100
T9	3	67 ± 31	384 ± 161

Of relevance, the series included one patient with normal ammonia (23 μmol/L, with a local laboratory threshold of 72 μmol/L) and two patients with subthreshold ammonia levels (69 and 70 μmol/L). These patients, despite not meeting the criteria for a diagnosis of OHE based on guidelines [1], were included because of their DRG discharge code (*please refer to* the *inclusion criteria in the Methods section*). Analyses were repeated having removed these three patients, and all results were confirmed.

## 4 Discussion

In this study, the time‐course of ammonia levels during a hospitalisation for OHE was assessed in relation to OHE duration/severity and the length of inpatient stay. Ammonia levels on admission correlated with the severity of the OHE episode, were significantly higher compared to both baseline and post‐discharge values, and significantly decreased over the first 24–36 h of hospitalisation. Higher OHE grades were associated to longer inpatient stays.

The choice of utilising the convenience period 2021–2024 is related to the fact that up until 2021 the COVID pandemic and its related rules still impinged on hospital admissions and their duration. The choice of including both Hepatic coma/HE as a first and as a second DRG code is related to the fact that in the latter instance the first DRG code was always cirrhosis, indicating that the main reason for admission was still OHE. Changes/choices in DRG order are generally related to consultants' preference, other DRG codes within the admission and changes in regional strategies for hospital financing over time.

Patients with a single compared to those with multiple hospitalisations for OHE were comparable in terms of demographic and clinical characteristics, including the aetiology and severity of liver disease, and OHE precipitants. This is in line with a study that investigated patients with recurrent OHE versus single episode OHE in a German cohort [16]. Unfortunately, the available information on the presence of spontaneous portal‐systemic shunts was limited in both groups in our study, and possibly insufficient to detect their expected higher prevalence in patients with multiple hospitalisations [7, 17]. It is also possible for the observation to be confounded by the selected time‐frame (i.e., a patient with a first episode in 2024 having a subsequent one in 2025, which will have not been detected).

All drugs administered to lower ammonia/ameliorate OHE symptoms were considered. Indeed, while non‐absorbable disaccharides and non‐absorbable antibiotics represent the cornerstone of OHE management [6, 18], additional therapies are available (e.g., enemas, laxatives other than non‐absorbable disaccharides [19, 20, 21], probiotics [22] and aminoacids [18]) and utilised in clinical practice even in the absence of RCTs and/or pertinent guidelines, particularly in patients with highly recurrent or persistent HE. The increased number of drugs to lower ammonia/ameliorate OHE symptoms prescribed on discharge compared to admission most likely reflects an attempt to reduce OHE recurrence, consistent with the above. In line with previously published literature, precipitants in most cases were mixed in our cohort [23]; the most common stand‐alone precipitants were dehydration, infection and constipation, again, in line with previously published literature [24].

Baseline/post‐discharge ammonia levels were significantly lower when compared to ammonia levels on admission. To our knowledge, this is the first study with available plasma ammonia levels in the same patient prior to, during and after an OHE‐related hospitalisation. These seem relevant to the debate on the usefulness of ammonia measurement [12, 13], which our findings, albeit preliminary and on a relatively small group of patients, would definitely support.

In line with previous literature [2, 3], in our study ammonia levels correlated with OHE severity. Ammonia levels significantly decreased within the first 24–36 h of hospitalisation, suggesting and confirming [6] that its monitoring in the first 1–2 days of hospitalisation may be useful, also to direct subsequent clinical decisions. However, given the retrospective nature of the study and measurements being available only in a subset of patients (92%), selection bias is likely, with ammonia measurement being repeated only/more frequently in patients whose neuropsychiatric status had not improved. By contrast, repeated ammonia measurement over the rest of the inpatient stay seemed of limited utility, as they remained substantially stable. Importantly, the role of blood ammonia reduction and its relationship with the symptoms over the course of an OHE‐related hospitalisation have not been fully elucidated. When plasma ammonia levels return to normal values, its cerebral consequences might lag for a certain time interval and thus the biochemical and clinical alterations may not match at all times. However, prospective and timed investigations are needed to confirm our findings and to better define the timing and usefulness of repeated ammonia measurement during a hospital stay, along with repeated assessment of mental status.

Finally, this study highlights how patients on chronic psychoactive medication (benzodiazepines, opioids or their derivatives, antidepressants, antipsychotics) and those discharged with residual OHE symptoms/signs had lower ammonia levels, suggesting that confounders may lower the relative increase in ammonia needed to result in the OHE phenotype expression. This is in line with a previous multicenter study in which patients were divided into four groups based on the presence/absence of acute‐on‐chronic liver failure (ACLF) and OHE [25]. The study showed that older patients with alcohol‐related aetiology developed HE in the absence of ACLF, suggesting that confounders/lower cognitive reserve [9] may play a role. Similarly, our findings confirm that patients who were discharged with residual neuropsychiatric symptoms/signs, most likely reflecting comorbidities, needed lower levels of ammonia to develop the OHE phenotype. The same applied to patients on chronic psychoactive treatment, which is in line with the role of drugs, psychoactive or otherwise (toxic and mixed encephalopathy), as reported by Desplats and co‐authors [10].

The study has a number of limitations. Data on the presence of portal‐systemic shunts, as well as biochemical data such as TSH, urinalyses, and procalcitonin were available in a small subset of patients. Further, a limited number of grade IV OHE records were included, possibly impinging on the value of observations made in this group. Most importantly, ammonia was measured based on the preference of the managing team rather than at fixed times. Nonetheless, the first ammonia measurement available after T0 was within 2–3 days in most instances and was shown to provide potentially useful information.

In conclusion, our study suggests that measuring ammonia during an acute OHE episode, both on admission and shortly after (most likely within 36 h), is likely to be useful. Further, similar but prospective and timed studies are probably worthwhile.

## Author Contributions

D.E. data acquisition and analysis, drafting of the manuscript. C.M. data analysis and review of the manuscript for important intellectual content. D.G. data acquisition. L.Z. data analysis and review of the manuscript for important intellectual content. S.M. study design and overview, data analysis, review of the manuscript for important intellectual content.

## Ethics Statement

Permission for retrospective data analysis was obtained from the local Ethics Committee.

## Conflicts of Interest

The authors declare no conflicts of interest.

## Supporting information


**Figure S1:** Ammonia levels (mean ±95% CI) prior to, on admission and after hospitalisation in patients grouped by resolution of OHE symptoms on discharge (panel A; resolution: *F* = 0.09, *p* = 0.76; time: *F* = 13.60, *p* < 0.0001; interaction: n.s.) and by chronic psychoactive medication (panel B; psychoactive medication: *F* = 4.10, *p* = 0.05; time: *F* = 7.03, *p* < 0.01; interaction: n.s.).

## Data Availability

The data that support the findings of this study are available on request from the corresponding author. The data are not publicly available due to privacy or ethical restrictions.
