# Supplementary figures and images for: Plasma Ammonia Levels Over the Course of a Hospitalisation for Overt Hepatic Encephalopathy
Source: Liver Int. 2025 Oct 3;45(11):e70365. doi: 10.1111/liv.70365 (PMC12494124; doi:10.1111/liv.70365)

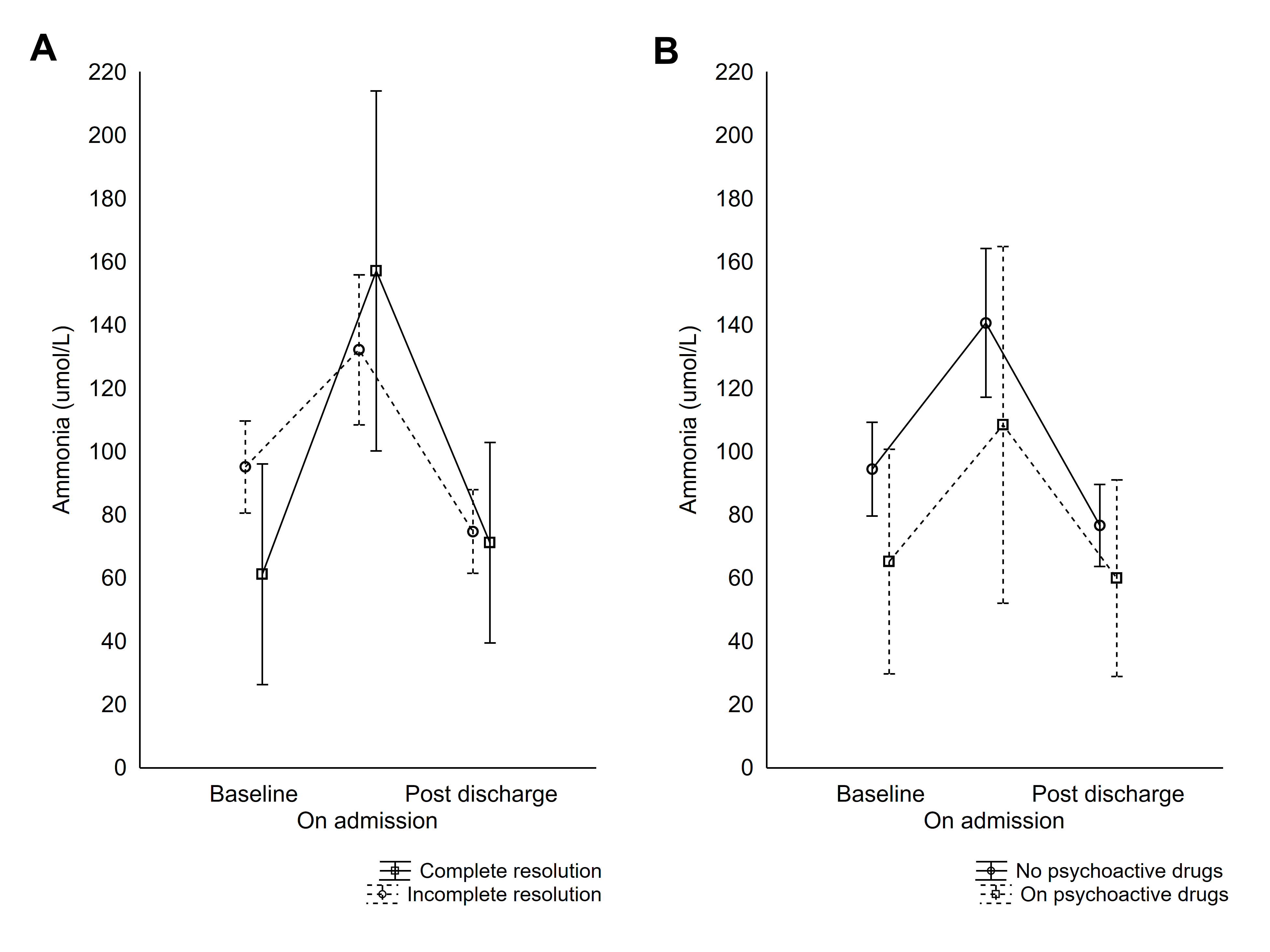

Supplement: Supplementary file 1 — Figure S1: Ammonia levels (mean ±95% CI) prior to, on admission and after hospitalisation in patients grouped by resolution of OHE symptoms on discharge (panel A; resolution: F = 0.09, p = 0.76; time: F = 13.60, p < 0.0001; interaction: n.s.) and by chronic psychoactive medication (panel B; psychoactive medication: F = 4.10, p = 0.05; time: F = 7.03, p < 0.01; interaction: n.s.). [file LIV-45-0-s001.tif]
